# Pervasive genetic associations between traits causing reproductive isolation in *Heliconius* butterflies

**DOI:** 10.1098/rspb.2010.1493

**Published:** 2010-09-01

**Authors:** Richard M. Merrill, Bas Van Schooten, Janet A. Scott, Chris D. Jiggins

**Affiliations:** 1 Department of Zoology, University of Cambridge, Downing Street, Cambridge CB2 3EJ, UK; 2 Department of Biology, University of Groningen, PO Box 14, 9750, AA, Haren, The Netherlands

**Keywords:** speciation, genetic linkage, mate choice, hybrid sterility, *Heliconius*

## Abstract

Ecological speciation proceeds through the accumulation of divergent traits that contribute to reproductive isolation, but in the face of gene flow traits that characterize incipient species may become disassociated through recombination. *Heliconius* butterflies are well known for bright mimetic warning patterns that are also used in mate recognition and cause both pre- and post-mating isolation between divergent taxa. Sympatric sister taxa representing the final stages of speciation, such as *Heliconius cydno* and *Heliconius melpomene*, also differ in ecology and hybrid fertility. We examine mate preference and sterility among offspring of crosses between these species and demonstrate the clustering of Mendelian colour pattern loci and behavioural loci that contribute to reproductive isolation. In particular, male preference for red patterns is associated with the locus responsible for the red forewing band. Two further colour pattern loci are associated, respectively, with female mating outcome and hybrid sterility. This genetic architecture in which ‘speciation genes’ are clustered in the genome can facilitate two controversial models of speciation, namely divergence in the face of gene flow and hybrid speciation.

## 1. Introduction

Whether disruptive selection can cause speciation in the face of gene flow remains a controversial problem in evolutionary biology [1]. Theoretical objections are typically based on the argument that hybridization and recombination will break down associations between traits that characterize emerging species [2]. Accordingly, genetic architectures that impede recombination can slow the breakdown of linkage disequilibrium and facilitate speciation [3]. For example, traits under disruptive ecological selection that are also used as mating cues can promote speciation because the same locus influences both assortative mating and ecological divergence (a ‘magic trait’ [4]). Similarly, chromosomal rearrangements such as inversions can prevent recombination between alleles [5,6] and may additionally permit the build-up of incompatibilities [7], despite gene flow in other parts of the genome. Tight linkage, and in some cases even the placement of isolating genes on the same chromosome, would act in a similar way [3,8]. Nonetheless, few empirical studies of animal taxa outside *Drosophila* have explored the genetic basis of both pre- and post-mating traits that contribute to reproductive and ecological isolation.

Butterfly wing patterns are often involved in ecological adaptation as well as mate choice [9,10], and are known to play a direct role in speciation [11,12]. *Heliconius* is a diverse neotropical genus famous for Müllerian mimicry, where unrelated species converge in their aposematic colour patterns to more efficiently advertise their unpalatability to predators. Sister taxa tend to belong to different mimicry rings and evidence suggests that shifts in colour pattern can cause both pre-mating and post-mating isolation, thereby promoting rapid speciation [13]. In at least five species, males prefer their own pattern over those of closely related taxa [13–18]. Colour pattern is also under strong frequency-dependent selection owing to predation, implying that recombinant, non-mimetic hybrids will be selected against [19]. These colour patterns are therefore a clear example of an ecological trait with a pleiotropic effect on mate choice and could be considered magic traits (*sensu* [4]).

Nonetheless, shifts in colour pattern must be accompanied by corresponding mate preferences to cause reproductive isolation. One possibility is that shifts in mate preference follow changes in colour pattern owing to selection for efficient mate finding. Hybrid zones between geographical races of *Heliconius melpomene* are in Hardy–Weinberg equilibrium, suggesting that divergent mate preferences observed between these varieties break down in the face of gene flow [14,20–22]. In later stages of speciation, however, there is evidence that hybridization may promote divergence through reinforcement or a similar process. Some parapatric taxa show bimodal hybrid zones [23,24], and sympatric species can show enhanced assortative mating in areas of contact [13,25]. Consistent with reinforcement, recombinant hybrids often experience decreased mating success [26] and female sterility [27,28], in addition to increased predation.

Where gene flow persists, speciation is facilitated by genetic linkage between traits that contribute to reproductive and ecological isolation. Examples of such associations have now been demonstrated for a handful of sympatric species [5,29–34], including one pair of parapatric *Heliconius* taxa, *Heliconius cydno* and *Heliconius pachinus*, where male preference and forewing colour map to a single quantitative trait locus (QTL) [15]. However, the generality of associations between loci for preference and preference cues, in addition to other traits that contribute reproductive and ecological isolation and their importance for speciation, remains unclear.

We study *H. cydno* and *H. melpomene*, species that are sympatric across much of central and northern South America. In addition to mimetic colour pattern and male preference, these species also differ in habitat and host–plant use, and these sister taxa probably represent the final stages of the speciation process [13,35,36]. Although hybrids between *H. cydno* and *H. melpomene* are rare in nature, gene flow persists at a low level [37,38] and fertile male hybrids can be produced in the insectary. We take advantage of this to explore the genetic basis of a suite of traits that contribute to reproductive isolation and reveal pervasive genetic associations between speciation genes.

## 2. Material and methods

### (a) Butterfly collection, crossing design and colour pattern scoring

Founder individuals of *Heliconius cydno chioneus* (CP) and *Heliconius melpomene rosina* (MP) were collected from Gamboa (9°7.4′ N, 79°42.2′ W, elevation 60 m) and the nearby Soberanía National Park, República de Panamá. Stock populations were kept in insectaries located in Gamboa. These were used to obtain F_1_ hybrids (CP mother and MP father) and backcross hybrids to each species (BC, male backcross to *H. cydno* and BM, male backcross to *H. melpomene*). Differences in wing colour pattern between *H. cydno* and *H. melpomene* are controlled by as few as 10 loci of major effect, seven of which are found on just two linkage groups [39]. This permits a simple QTL analysis using segregation of phenotypic traits as genetic markers. Wings were scored for colour pattern loci following Naisbit *et al*. [39]: BC individuals were scored at the *B*, *Ac* and *Yb* loci (which are unlinked) and BM individuals at the *Yb* locus (see the electronic supplementary material, figure S1). Although further colour pattern loci also segregate in BC and BM individuals, these could not be scored with confidence owing to wing wear and were not included in our analysis. BC individuals heterozygous at the *B* locus (*Bb*) have a red forewing band (as observed in *H. melpomene*), whereas homozygous individuals (*bb*) lack this (both genotypes also have a white forewing band controlled by a different locus [39]). The *Ac* locus also segregates in BC individuals resulting in the presence (*acac*) or absence (*Acac*) of the white hourglass shape observed in the main forewing cell of *H. cydno* [39]. The partially dominant *Yb* locus, which controls the expression of the yellow hindwing bar seen in *H. melpomene rosina* (*ybyb*), segregates in BC individuals so that heterozygous individuals (*Ybyb*) show a shadow of the bar formed by melanic scales with altered reflectance and can be distinguished from homozygous individuals (*YbYb*) [39].

In addition to scoring individuals for colour pattern loci, we also genotyped a subset of individuals across a panel of eight unlinked molecular markers (electronic supplementary material, table S1). This resulted in a reduced dataset of between 37 and 43 males and between 38 and 44 females for which we had tissue and were confidently able to assign the ancestry of alleles. Of the eight markers used, seven have previously been mapped [40,41] to different linkage groups that do not contain *B*, *Ac* or *Yb*. The eighth marker, *Ci*, is on the same the linkage group as the *B* locus; however, *Ci* is known to be approximately 65 cm from *B* [42] and in our broods, there was no significant association between the two loci (*p* > 0.1), implying high rates of recombination between these two loci. These markers serve as a control in testing for genetic associations between traits that contribute to reproductive isolation.

### (b) Male preference experiments

We determined individual male mate preferences for *H. cydno chioneus* (CP) and *H. melpomene rosina* (MP), their F_1_ hybrids (F_1_) and backcross hybrids to each species. Males were introduced into experimental cages (1 × 1 × 2 m) with a virgin female of each species (0–10 days matched for age). Female pairs were re-used and replaced when fresh individuals became available. Fifteen-minute trials were divided into 1 min intervals, which were scored for courtship (sustained hovering or chasing) directed towards each female as having occurred (1) or not occurred (0). Accordingly, if a male courted the same female twice within a minute interval, it was recorded only once; if courtship continued into a second minute, it was recorded twice. On mating, couples were rapidly and gently separated; this does not affect subsequent behaviours [13]. Where possible, trials were repeated five times for each male, producing individual scores of total courtships and mating attempts directed towards females of each species.

Models for our second male preference experiment were made from dissected *H. cydno* and *H. melpomene* wings. After removal, wings were washed for 5 min in hexane to remove cuticular hydrocarbons. Models, one made from the wings of each species and attached to equal lengths of flexible wire, were presented simultaneously to males in a 2 × 2 × 2 m insectary. Models were manipulated to simulate flight and males were tested individually in 5 min trials during which the number of courtships (sustained hovering) directed towards each model was recorded. Trials were repeated five times for each male.

### (c) Female ‘choice’ experiment

Twenty *H. cydno* and 20 *H. melpomene* males were maintained in an experimental cage (3 × 5 × 3 m) with ample pollen sources as well as artificial nectar. Virgin females were introduced once they were able to fly (normally 1–2 h after eclosion) and removed on mating or after 72 h if they remained unmated. Because pairs stay coupled for at least 1 h, we were able to record all matings by monitoring the experimental cage every hour between 7.00 and 18.00 h. After mating, males were replaced with fresh individuals from stock populations.

To quantify relative male interest, we recorded courtship events by *H. cydno* and *H. melpomene* males during 10 min focal observations of individual females. As with our live-female male preference experiment, observations were divided into 1 min intervals during which courtships by each species were scored as having occurred (1) or not having occurred (0). In this case, courtship events could be scored multiple times during each minute interval when performed by different males. Observations were paused if the female flew out of sight and restarted when she was relocated.

### (d) Female sterility experiment

*Passiflora menispermifolia* and *Passiflora vitifolia*, with fresh shoots for oviposition, were provided to individual mated females. Eggs were collected and numbers recorded daily. We considered mated hybrid females as sterile once two criteria had been met: first, females that did not lay eggs had been in the cage for a minimum of 8 days; second, females that did not lay eggs were at least 15 days old. Non-laying females that did not meet these criteria were excluded from analysis.

### (e) Statistical analysis

Using the data collected in our male preference experiments, we estimated relative probabilities of male courtship directed towards *H. melpomene* rather than *H. cydno* females or models with likelihood [13,15,43]. The likelihood function was
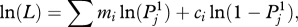

where *m*_*i*_ is the total number of courtship events by male *i* directed towards *H. melpomene, c*_*i*_ the total number of events by male *i* directed towards *H. cydno*, and 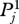 the probability of males of genotype *j* performing behaviours directed towards *H. melpomene*. Probabilities of male courtship were estimated by numerically searching for values of 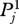 that maximized ln(*L*), using the solver option in Excel (Microsoft). Support limits, asymptotically equivalent to 95 per cent confidence intervals, were obtained by searching for values that decreased ln(*L*) by two units [43].

Essentially the same likelihood function and approach was used to analyse the male interest data collected in our female experiment; however, in this case, *m*_*i*_ and *c*_*i*_ are the total number of courtship events directed towards female *i* by *H. melpomene* and *H. cydno* males, respectively; similarly, for these data, 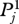 is the probability of *H. melpomene* males performing behaviours directed towards females of genotype *j*.

To compare individuals that differed at colour pattern and molecular marker loci, we first produced a model where relative probabilities for different genotypes were set equal (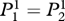). This was then compared with a second model in which relative probabilities for each genotype were estimated separately (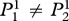) using a likelihood ratio test with *G* = 2Δln(*L*), which asymptotically follows a *χ*^2^ distribution with one degree of freedom. Reported *p*-values are after Bonferroni correction for multiple comparisons where appropriate.

We used generalized linear models (GLMs) with binomial error distribution to analyse data collected in our female mating and female sterility experiments. To test for additive effects of colour pattern chromosomes, models were first fitted with all loci as explanatory variables. In all cases, model simplification resulted in a minimum adequate model with a single explanatory variable, and significance was tested with *χ*^2^-tests. Owing to the small number of individuals for which we had sterility data and were able to genotype at our molecular control loci, we were unable to fit a binomial GLM for this analysis. Instead, we tested for associations between molecular control loci and the failure to lay eggs using Fisher's exact tests, with a Bonferroni correction across the nine tests.

## 3. Results

### (a) Male preference for wing colour pattern is linked to forewing coloration

**Figure 1.**
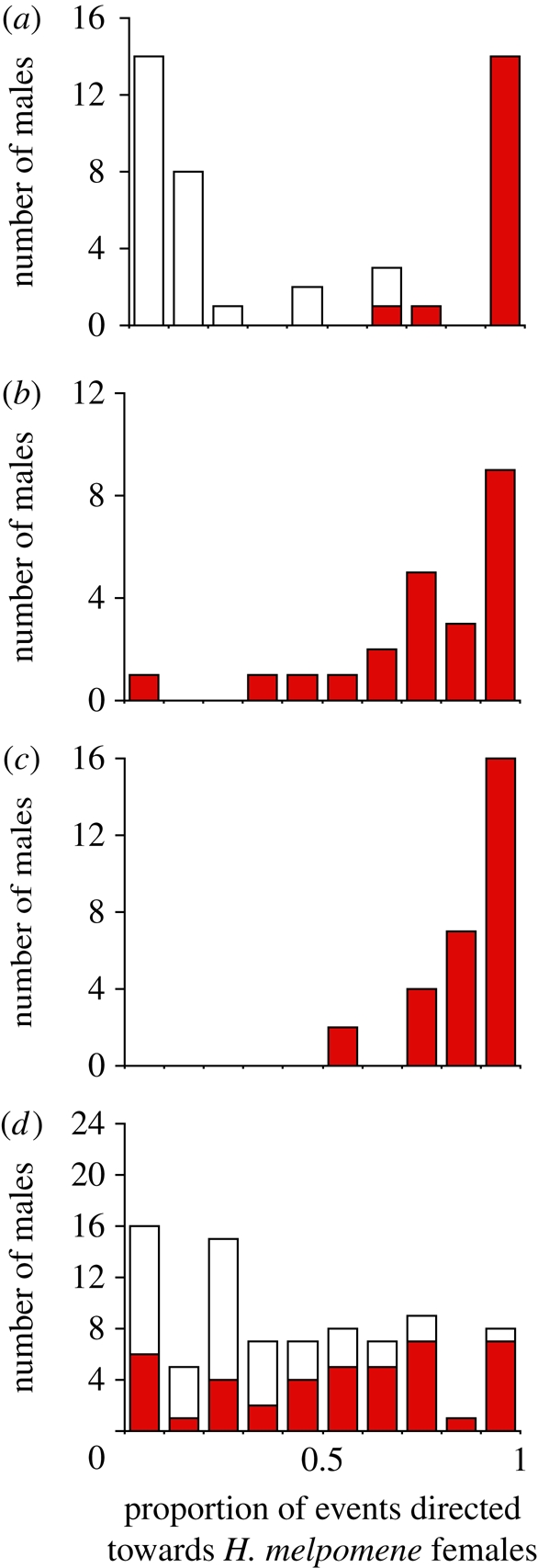
Male mate preference of *H. cydno*, *H. melpomene* and their hybrids. Proportion of courtship events directed towards live *H. melpomene* females by (*a*) *H. cydno* (white bars) and *H. melpomene* (red bars) males; (*b*) their F_1_ hybrid males; and male offspring from backcross broods to each species; (*c*) backcross to *H. melpomene* and (*d*) backcross to *H. cydno*. In each case, red bars represent individuals with a red forewing bar (*BB* or *Bb*) and white bars individuals without the red forewing bar (*bb*).

In total, we recorded 1814 courtship events by 183 males in 856 15 min trials. Data for CP and MP males have been previously published elsewhere, but we include them here for comparison. As expected, the probabilities of courtship were predicted by the genetic contribution of the parental species (i.e. CP < BC < F_1_ < BM < MP; electronic supplementary material, table S2). CP and MP preferred conspecific females, whereas F_1_ males displayed a more intermediate preference but with a considerable skew towards *H. melpomene* (figure 1*a*,*b*). Very little segregation in mate preference was observed among BM males and as a group, they differed little from MP (figure 1*c*). By contrast, BC males showed the full range of mate preference (figure 1*d*).

**Figure 2.**
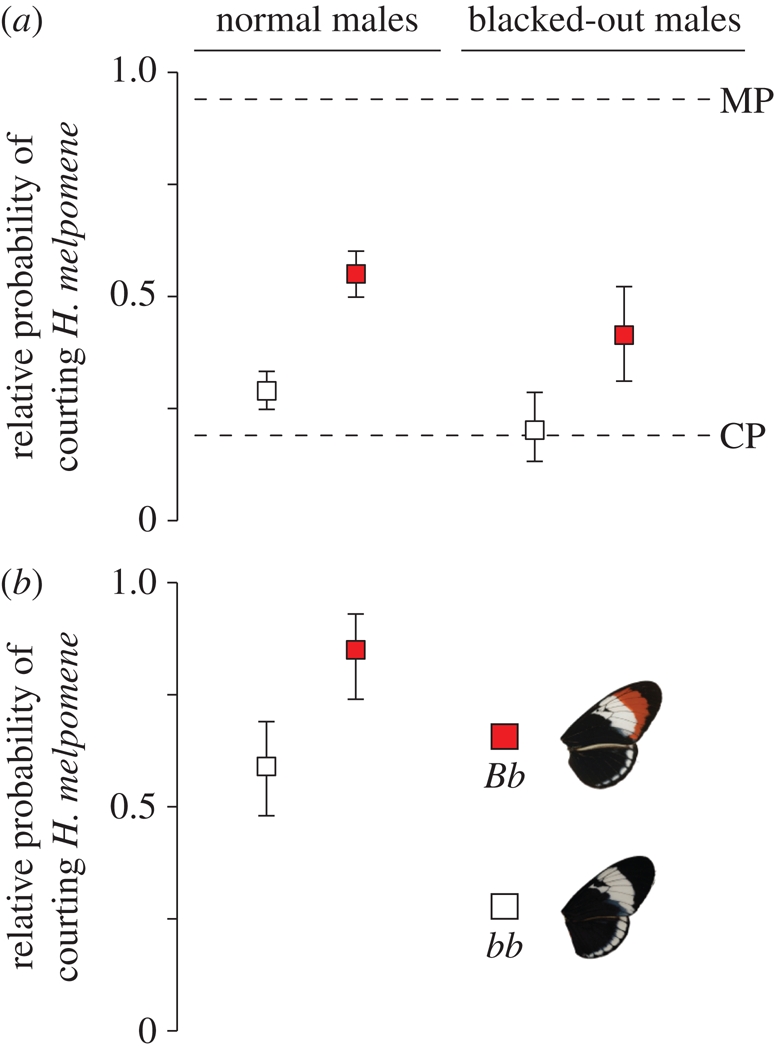
Male mate preference is associated with forewing colour. The probability of courting *H. melpomene* live females (*a*) and wing pattern models (*b*) by backcross hybrids to *H. cydno* (BC) that have the red forewing band (*Bb*, red squares) and those that do not (*bb*, white squares), where 1 would indicate a complete preference for *H. melpomene* and 0 a preference for *H. cydno*. Blacked-out males had their forewing colour pattern obscured in order to prevent self-matching. Dashed lines represent the probabilities of courting live *H. melpomene* females for *H. melpomene* (MP) and *H. cydno* (CP) males. Support limits are asymptotically equivalent to 95% confidence intervals and were obtained by searching for values that decreased ln(*L*) by two units.

Individuals with the red forewing band (*Bb*) were more likely to court *H. melpomene* rather than *H. cydno* females as compared with homozygous individuals (*bb*) (*G* = 58.64, *p*<< 0.001; figure 2*a*). Our probability estimates for the two *B* locus genotypes, in addition to those for CP and MP males (electronic supplementary material, tables S2 and S3A), reveal effect sizes for the *B* locus of 34.7 per cent of the measured differences in courtship between *H. melpomene* and *H. cydno*. After Bonferroni correction for multiple comparisons, neither variation at the *Ac* locus nor at the *Yb* locus was significantly associated with male preference for live females. Similarly, there were no significant associations between any of our molecular control markers and male mate preference (electronic supplementary material, table S4A). With the exception of the microsatellite marker *Hm02* on linkage group 3, the association between male mate preference and the *B* locus remained significant despite the reduced number of individuals that we were able to score at molecular markers (electronic supplementary material, table S4B). Consequently, the lack of additional associations does not appear to be owing to a lack of statistical power.

A previous study of races of *H. melpomene* found no experimental evidence for learning of male preferences from other individuals [14] and the hybrids used here were kept in mixed male-only groups. Nonetheless, our results might also be explained by self-matching, so we repeated the experiment with a further 17 BC males that eclosed in the dark and then had their forewing band blacked-out using a *Sharpie* marker pen under red light. Males heterozygous at the *B* locus were still more likely to court *H. melpomene* than *H. cydno* females as compared with homozygous individuals (*G* = 10.44, *p* < 0.005; figure 2*a*) even though they did not have the opportunity to learn their own colour pattern.

To investigate the cues used in male mate choice, we tested 42 BC males in preference trials with models made from *Heliconius* wings (washed in hexane to remove cuticular hydrocarbon cues). Once again, individuals heterozygous at the *B* locus were more likely to court the *H. melpomene* pattern (*G* = 11.42, *p* < 0.005; figure 2*b*), demonstrating that the preference was at least in part for colour pattern, as opposed to other pheromonal or behavioural cues. Again, we found no significant differences associated with either the *Ac* or *Yb* loci (electronic supplementary material, table S3B).

### (b) Female mating outcome and male interest

**Figure 3.**
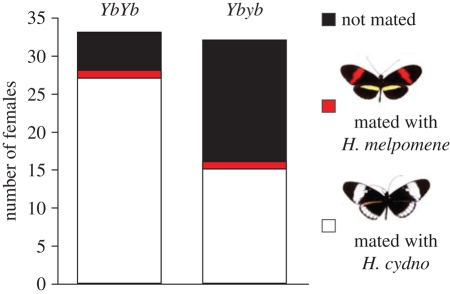
Female mate choice is associated with the hindwing *Yb* colour pattern locus. Data are counts of female offspring from backcross to *H. cydno* broods (BC) differing at the *Yb* locus that mated with *H. cydno* (white), *H. melpomene* (red) or did not mate (black) within 3 days of introduction to an experimental cage containing 20 males of each species.

Overall, in our female choice experiment, there was a high failure to mate among hybrid females, consistent with a previous study of F_1_ hybrids [26], and variation at the *Yb* locus was strongly associated with propensity to mate among BC females. Under the same conditions, six individual MP and four CP females were all rapidly mated by males of their own species, while of 36 F_1_ females, 25 remained unmated after 3 days (four mated with *H. cydno*, seven mated with *H. melpomene*). Among backcross females, 62 of 98 BC females of all genotypes mated with *H. cydno* and only four mated with *H. melpomene*; similarly, of 29 BM females, 21 mated with *H. melpomene* and none mated with *H. cydno*. Those BC females that inherited an allele from *H. melpomene* at the *Yb* locus were much less likely to mate (*p* < 0.005; figure 3), while there was no significant effect of either the *B* or *Ac* loci. Analysis of BC females scored at molecular markers revealed no further significant associations with female mating outcome, and individuals heterozygous at the *Yb* locus were still less likely to mate (*p* < 0.05) despite the reduced dataset. As the failure to mate represents a breakdown of parental behaviour, we cannot use the difference between parental values to estimate the strength of the *Yb* locus effect. However, using the full dataset, the *Yb* locus explained 12 per cent of the variance in mating outcome among BC females.

Our focal observations revealed that among BM females, *H. melpomene* males were more likely to court individuals homozygous at the *Yb* locus, expressing the *H. melpomene* yellow hindwing bar (*ybyb*), compared with those heterozygous at this locus (*G* = 4.09, *p* < 0.05). The *Yb* locus segregates with the *N* locus, which when heterozygous in BM individuals reveals a white or yellow forewing band in addition to the red [39]. Differences in male interest between *Yb* genotypes are therefore likely to be owing to forewing colour pattern.

While *H. cydno* males courted *Bb* and *bb* BC females equally, *H. melpomene* males were seven times more likely to court females with the red band (*Bb*), with the relative probabilities of courtship (*G* = 27.84, *p*<< 0.001) differing significantly with respect to the female genotype at this locus. By contrast, we found no effect of either the *Ac* or *Yb* loci on male interest for BC females (electronic supplementary material, table S4). The *Yb* genotype of BC females therefore had no observed effect on male interest, but a significant association with female mating outcome (see above), which is most readily explained as an effect of this locus on female choice possibly through mate rejection behaviour.

### (c) Female hybrid sterility is associated with colour pattern

**Figure 4.**
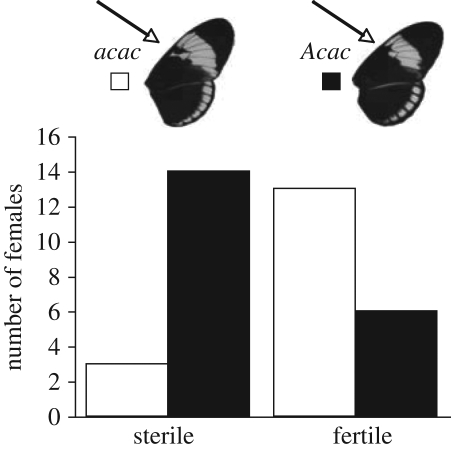
Failure to oviposit is associated with the *Ac* forewing colour pattern locus. White bars represent female offspring from backcross to *H. cydno* broods (BC) that are homozygous at the locus (*acac*) for the *H. cydno* allele and black bars represent heterozygous females at the locus (*Acac*), having inherited an allele from both *H. cydno* and *H. melpomene*. Data are counts of females that failed to lay eggs.

As expected, all CP (*n* = 13) and MP (*n* = 21) females tested laid eggs, and of 20 F_1_ females tested, 14 laid eggs. With the exception of two individuals, all BM females (*n* = 18) laid eggs. Among BC females, there was clear segregation of behavioural sterility, and those that inherited an allele from *H. melpomene* at the *Ac* locus (*Acac*) more often failed to lay eggs after 8 days (*p* < 0.005; figure 4), implying sterility associated with this locus. Segregation of the *Ac* locus explained 20 per cent of the variance in oviposition probability of BC females. In contrast to our molecular control loci, the *Ac* retained a significant association with the failure of BC females to lay eggs after Bonferroni correction for multiple comparisons (electronic supplementary material, table S5).

## 4. Discussion

Genetic architecture can profoundly influence the process of speciation, but empirical studies have focused on the genetics of intrinsic hybrid inviability and sterility rather than behavioural traits. Our experimental results demonstrate pervasive genetic associations between ecologically relevant traits that contribute to both pre- and post-mating isolation. Male preference and female mating outcome are associated with two linkage groups that are already known to contain seven of the 10 major colour pattern loci that distinguish *H. cydno* and *H. melpomene* (male preference is associated with the Br-B-G linkage group and female mating outcome is associated with the N-Sb-Vf-Yb linkage group [39]). In addition, loci affecting female hybrid sterility are associated with a further colour pattern locus situated on a third chromosome. Thus, our study is significant in reporting physical linkage between multiple ‘speciation genes’ that are localized with respect to the rest of the genome.

Linkage between preference and colour pattern was strongest at the *B* locus, which controls forewing coloration. Not only were males with the red forewing band more likely to court *H. melpomene* females, but our model experiments confirm that the preference segregating with the *B* locus is, at least in part, for colour pattern rather than other behavioural or pheromonal cues. Furthermore, these differences account for a large proportion of the measured phenotypic difference between the parental species (34.7%), implying that while there may be other unmapped loci that affect male preference, the QTL at the *B* locus is of major effect. Kronforst *et al*. [15] have shown a similar association between yellow/white forewing coloration and male mate preference for live females in two parapatric members of the *cydno* clade, *Heliconius cydno galanthus* and *H. pachinus*, as well as in the polymorphic Ecuadorian race *Heliconius cydno alithea* [15,44]. The loci studied here are on different chromosomes, such that there is now strong evidence for genetic associations between trait and male preference loci on at least two distinct chromosomes.

Female *Heliconius* can mate soon after eclosion before their wings have fully inflated and when there is little opportunity to fend off undesirable suitors, contributing to an assumption that assortative mating is largely a result of male choice (e.g. [13]). However, in our experiments, BC females with different *Yb* genotypes were courted equally by males, but showed a significant difference in mating outcome. We waited until females could fly before introducing them to our experimental cage, such that they were able to reject males by spreading their wings and raising their abdomen out of the reach of male claspers. Thus, the simplest explanation for the data is that the *Yb* genotype influences female choice, although we cannot completely rule out cryptic male choice that is not reflected in our observations of male behaviour. Nonetheless, to our knowledge, this study represents the first evidence for female choice in *Heliconius* and emphasizes the potential importance it may have for speciation.

We have also provided novel evidence for sterility associated with a third wing-patterning locus. Previous studies, including one involving the same races studied here, have demonstrated a large Z-chromosome effect on sterility, and in particular a strong association between the sex-linked gene *Tpi* and hatch rate [27,28,45]. Naisbit *et al.* [28] state that in the backcross to *H. melpomene*, ‘sterile females typically laid eggs that did not hatch’ and sterility was associated with *Tpi*. By contrast, ‘in the backcross to *H. cydno*, complete sterility was usually manifested as a failure to lay eggs’, and in this cross there was no evidence of a large Z-chromosome effect. Here, we demonstrate that female sterility is associated with *Ac*, an autosomal locus.

Without additional molecular markers, our data do not permit precise genomic mapping of QTL; nonetheless, *H. cydno* and *H. melpomene* have 21 pairs of chromosomes [46] and high rates of recombination [40], such that the association of loci affecting reproductive isolation is very unlikely by chance alone [15]. The lack of association between traits contributing to reproductive isolation and any of our eight molecular control loci further supports this. The effect of the *B* locus on hybrid male mate preference explains over 30 per cent of the difference between parental species in mate preference. For the other traits, which involve breakdown of parental behaviours, we used the percentage of phenotypic variance explained by colour pattern genotype to estimate the size of the effect. The variance between individuals obviously includes an environmental component, which we are unable to quantify and which is likely to be large for behavioural experiments conducted in outdoor insectaries. Thus, our estimates of the percentage of phenotypic variance explained are a very conservative estimate of the effect size of the loci studied. Despite this, the percentage of phenotypic variance explained varied from 12 per cent for the effect of *Yb* on female mating outcome to 20 per cent for the *Ac* locus effect on female sterility.

Our study contributes to a handful of examples where genetic architecture reduces recombination between traits that contribute to reproductive isolation. For example, in two species of European flycatcher, genes that influence female preference, male plumage and reduced hybrid fitness have been shown to be Z-linked [32,33]; however, theory also predicts the build-up of speciation genes on the sex chromosomes for reasons unrelated to persistent gene flow, as reviewed by Qvarnströn & Bailey [47]. Additional studies have shown autosomal associations between a mating trait and mate preference, notably in Hawaiian crickets and a previous study on *Heliconius* [15,34,44]. Similarly, in two host races of the pea aphid, there is a genetic association between host preference and host-associated performance, which would act to facilitate ecological speciation [29].

These examples involve associations between just one pair of traits at a single QTL, but ecological speciation probably proceeds through the accumulation of multiple species-specific adaptations. Perhaps the best comparable study of multiple traits involved in speciation, therefore, involves the hybridizing species *Drosophila pseudoobscura* and *Drosophila persimilis*, where virtually all species differences are controlled by genes in fixed inversions between the species [5,30,48,49]. In that case, the inversions account for a large proportion of the four chromosomes making up the *Drosophila* genome, while by contrast, our results show localization of genes to three chromosomal regions, representing a much smaller proportion of the genome (3/21 chromosomes). Around the *B* locus, there is evidence for reduced recombination across a region of 700 kb, which might result from a local inversion polymorphism segregating in *H. melpomene* [42]. Currently, we cannot determine whether the associations between the loci studied here are owing to pleiotropy, tight linkage, inversion polymorphism or some other mechanism by which recombination is suppressed. In contrast to *Drosophila*, because Lepidoptera have a large number of chromosomes, even if our trait loci map to inversions, they would still be free to recombine with respect to the majority of the genome.

Whatever the genetic and evolutionary mechanisms responsible, linkage between colour pattern and preference loci is likely to have been important during the adaptive radiation of *Heliconius*. In diverging populations, linkage will strongly reduce recombination between mimetic wing patterns and mate choice, thereby simultaneously promoting ecological and reproductive isolation [15]. There are two possible explanations for the observed association. First, allopatric divergence may have been followed by contact with gene flow homogenizing species differences across much of the genome and leaving only those differences that have accumulated in tightly linked regions. Alternatively, divergence in sympatry could have involved species differences accumulating preferentially in linked regions. Regardless of whether or not there has been allopatry in the past, our results reveal a genetic architecture that facilitates divergence in the face of gene flow.

Such associations could also promote hybrid trait speciation [50]. Several putative examples of hybrid species in *Heliconius* are proposed to have derived from interbreeding between *H. cydno* and *H. melpomene*. Furthermore, the forewing of the best-supported example, the Colombian species *Heliconius heurippa* [16], involves both yellow and red pattern elements controlled by the *N* and *B* loci, which we show to be linked to loci for female and male preference, respectively (*N* is tightly linked to *Yb*). Indeed, a recent study [51] reveals that *H. heurippa*-like males, reconstructed by backcrossing F_1_ hybrids into *H. cydno*, are more likely to approach and court models of their own colour pattern than either of the parental species, suggesting that hybridization could very rapidly lead to pre-mating isolation. Our results provide a mechanism by which the introgression of colour pattern elements would directly lead to assortative mating through linkage of the corresponding preference alleles. This offers a potential mechanism for rapid hybrid speciation. Overall, our results show clustering of ‘speciation genes’ and add to a growing body of evidence that genetic architecture can facilitate speciation in the face of gene flow.

## References

[1] Coyne J. A. Orr H. A. 2004 Speciation. Sunderland, MA: Sinauer

[2] Mayr E. 1963 Animal species and evolution. Cambridge, MA: BelKnap

[3] Servedio M. R. 2009 The role of linkage disequilibrium in the evolution of premating isolation. Heredity 102, 51–56 10.1038/hdy.2008.98 (doi:10.1038/hdy.2008.98) 18813328

[4] Gavrilets S. 2004 Fitness landscapes and the origin of species. Princeton, NJ: Princeton University Press

[5] Noor M. A. F. Grams K. L. Bertucci L. A. Reiland J. 2001 Chromosome inversions and the reproductive isolation of species. Proc. Natl Acad. Sci. USA 98, 12 084–12 088 10.1073/pnas.221274498 (doi:10.1073/pnas.221274498) PMC59771 11593019

[6] Rieseberg L. H. 2001 Chromosomal rearrangements and speciation. Trends Ecol. Evol. 16, 351–358 10.1016/S0169-5347(01)02187-5 (doi:10.1016/S0169-5347(01)02187-5) 11403867

[7] Navarro A. Barton N. H. 2003 Accumulating postzygotic isolation genes in parapatry: a new twist on chromosomal speciation. Evolution 57, 447–459 12703935 10.1111/j.0014-3820.2003.tb01537.x

[8] Felsenstein J. 1981 Skepticism towards Santa Rosalia, or why are there so few kinds of animals? Evolution 35, 124–138 10.2307/2407946 (doi:10.2307/2407946) 28563447

[9] Heinrich B. 1993 The hot-blooded insects: strategies and mechanisms of thermoregulation. Cambridge, MA: Harvard University Press

[10] Nijhout H. 1991 The development and evolution of butterfly wing patterns. Washington (DC): Smithsonian Institution Press

[11] Bates H. W. 1862 Contributions to an insect fauna of the Amazon valley (Lepidoptera: Heliconidae). Trans. Linn. Soc. Lond. 23, 495–566 10.1111/j.1096-3642.1860.tb00146.x (doi:10.1111/j.1096-3642.1860.tb00146.x)

[12] Vane-Wright R. I. 1978 Ecological and behavioural origins of diversity in butterflies. In Diversity of insect faunas, vol. 9 (eds Mound L. Waloff N.), pp. 56–70 Oxford, UK: Blackwell Scientific

[13] Jiggins C. D. Naisbit R. E. Coe R. L. Mallet J. 2001 Reproductive isolation caused by colour pattern mimicry. Nature 411, 302–305 10.1038/35077075 (doi:10.1038/35077075) 11357131

[14] Jiggins C. D. Estrada C. Rodrigues A. 2004 Mimicry and the evolution of premating isolation in *Heliconius melpomene* Linnaeus. J. Evol. Biol. 17, 680–691 10.1111/j.1420-9101.2004.00675.x (doi:10.1111/j.1420-9101.2004.00675.x) 15149410

[15] Kronforst M. R. Young L. G. Kapan D. D. McNeely C. O'Neil R. J. Gilbert L. E. 2006 Linkage of butterfly mate preference and wing color preference at the genomic location *wingless*. Proc. Natl Acad. Sci. USA 103, 6575–6580 10.1073/pnas.0509685103 (doi:10.1073/pnas.0509685103) 16611733 PMC1458925

[16] Maváraz J. Salazar C. A. Bermingham E. Salcedo C. Jiggins C. D. Linares M. 2006 Speciation by hybridization in *Heliconius* butterflies. Nature 441, 868–871 16778888 10.1038/nature04738

[17] Estrada C. Jiggins C. D. 2008 Interspecific sexual attraction because of convergence in warning colouration: is there a conflict between natural and sexual selection in mimetic species? J. Evol. Biol. 21, 749–760 10.1111/j.1420-9101.2008.01517.x (doi:10.1111/j.1420-9101.2008.01517.x) 18312559

[18] Muñoz A. G. Salazar C. Castaño J. Jiggins C. D. Linares M. 2010 Multiple sources of reproductive isolation in a bimodal butterfly hybrid zone. J. Evol. Biol. 23, 1312–1320 10.1111/j.1420-9101.2010.02001.x (doi:10.1111/j.1420-9101.2010.02001.x) 20456567

[19] Mallet J. Barton N. 1989 Strong natural selection in a warning color hybrid zone. Evolution 43, 421–431 10.2307/2409217 (doi:10.2307/2409217) 28568556

[20] Mallet J. 1986 Hybrid zones in *Heliconius* butterflies in Panama, and the stability and movement of warning colour clines. Heredity 56, 191–202 10.1038/hdy.1986.31 (doi:10.1038/hdy.1986.31)

[21] Mallet J. 1993 Speciation, raciation, and color pattern evolution in *Heliconius* butterflies: evidence from hybrid zones. In Hybrid zones and the evolutionary process (ed. Harrison R. G.), pp. 226–260 New York, NY: Oxford University Press

[22] Turner J. R. G. 1971 Two thousand generations of hybridization in a *Heliconius* butterfly. Evolution 25, 471–482 10.2307/2407345 (doi:10.2307/2407345) 28565031

[23] Arias C. F. Muñoz A. G. Jiggins C. D. Maváraz J. Bermingham E. Linares M. 2008 A hybrid zone provides evidence for incipient ecological speciation in *Heliconius* butterflies. Mol. Ecol. 17, 4699–4712 10.1111/j.1365-294X.2008.03934.x (doi:10.1111/j.1365-294X.2008.03934.x) 18828780

[24] Jiggins C. D. McMillan W. O. Neukirchen W. Mallet J. 1996 What can hybrid zones tell us about speciation? The case of *Heliconius erato* and *H. himera* (Lepidoptera: Nymphalidae). Biol. J. Linn. Soc. 59, 221–241

[25] Kronforst M. R. Young L. G. Gilbert L. E. 2007 Reinforcement of mate preference among hybridizing *Heliconius* butterflies. J. Evol. Biol. 20, 278–285 10.1111/j.1420-9101.2006.01198.x (doi:10.1111/j.1420-9101.2006.01198.x) 17210020

[26] Naisbit R. E. Jiggins C. D. Mallet J. 2001 Disruptive sexual selection against hybrids contributes to speciation between *Heliconius cydno* and *Heliconius melpomene*. Proc. R. Soc. Lond. B 268, 1849–1854 10.1098/rspb.2001.1753 (doi:10.1098/rspb.2001.1753) PMC1088818 11522205

[27] Jiggins C. D. Linares M. Naisbit R. E. Salazar C. A. Yang Z. H. Mallet J. 2001 Sex-linked hybrid sterility in a butterfly. Evolution 55, 1631–1638 11580022 10.1111/j.0014-3820.2001.tb00682.x

[28] Naisbit R. E. Jiggins C. D. Linares M. Salazar C. Mallet J. 2002 Hybrid sterility, Haldane's rule and speciation in *Heliconius cydno* and *H. melpomene*. Genetics 161, 1517–1526 12196397 10.1093/genetics/161.4.1517 PMC1462209

[29] Hawthorne D. J. Via S. 2001 Genetic linkage of ecological specialization and reproductive isolation in pea aphids. Nature 412, 904–907 10.1038/35091062 (doi:10.1038/35091062) 11528477

[30] Noor M. A. F. Grams K. L. Bertucci L. A. Almendarez Y. Reiland J. Smith K. 2001 The genetics of reproductive isolation and the potential for gene exchange between *Drosophila pseudoobscura* and *D. persimilis* via backcross hybrid males. Evolution 55, 512–521 10.1554/0014-3820(2001)055[0512:TGORIA]2.0.CO;2 (doi:10.1554/0014-3820(2001)055[0512:TGORIA]2.0.CO;2) 11327159

[31] Rieseberg L. H. Whitton J. Gardner K. 1999 Hybrid zones and the genetic architecture of a barrier to gene flow between two sunflower species. Genetics 152, 713–727 10353912 10.1093/genetics/152.2.713 PMC1460641

[32] Sæther S. A. 2007 Sex chromosome-linked species recognition and evolution of reproductive isolation in flycatchers. Science 318, 95–97 10.1126/science.1141506 (doi:10.1126/science.1141506) 17916732

[33] Sæthre G. Borge T. Lindroos K. Haavie J. Sheldon B. C. P. Syvänen A. 2003 Sex chromosome evolution and speciation in Ficedula flycatchers. Proc. R. Soc. Lond. B 270, 53–59 10.1098/rspb.2002.2204 (doi:10.1098/rspb.2002.2204) PMC1691206 12590771

[34] Shaw K. L. Lesnick S. C. 2009 Genomic linkage of male song and female acoustic preference QTL underlying a rapid species radiation. Proc. Natl Acad. Sci. USA 106, 9737–9742 10.1073/pnas.0900229106 (doi:10.1073/pnas.0900229106) 19487670 PMC2701026

[35] Estrada C. Jiggins C. D. 2002 Patterns of pollen feeding and habitat preference among *Heliconius* species. Ecol. Entomol. 27, 448–456 10.1046/j.1365-2311.2002.00434.x (doi:10.1046/j.1365-2311.2002.00434.x)

[36] Smiley J. 1978 Plant chemistry and the evolution of host specificity: new evidence from *Heliconius* and *Passiflora*. Science 201, 745–747 10.1126/science.201.4357.745 (doi:10.1126/science.201.4357.745) 17750235

[37] Bull V. Beltrán M. Jiggins C. D. McMillan W. O. Bermingham E. Mallet J. 2006 Polyphyly and gene flow between non-sibling *Heliconius* species. BMC Biol. 4, 11 10.1186/1741-7007-4-11 (doi:10.1186/1741-7007-4-11) 16630334 PMC1481601

[38] Kronforst M. R. Young L. Blume L. Gilbert L. E. 2006 Multilocus analysis of admixture and introgression among *Heliconius* butterflies. Evolution 60, 1254–1268 16892975

[39] Naisbit R. E. Jiggins C. D. Mallet J. 2003 Mimicry: developmental genes that contribute to speciation. Evol. Dev. 5, 269–280 12752766 10.1046/j.1525-142x.2003.03034.x

[40] Jiggins C. D. Mavarez J. Beltrán M. McMillan W. O. Johnston J. S. Bermingham E. 2005 A genetic linkage map of the mimetic butterfly *Heliconius melpomene*. Genetics 171, 557–570 10.1534/genetics.104.034686 (doi:10.1534/genetics.104.034686) 15489522 PMC1456771

[41] Pringle E. G. Baxter S. W. Webster C. L. Papanicolaou A. Lee S. F. Jiggins C. D. 2007 Synteny and chromosome evolution in the Lepidoptera: evidence from mapping in *Heliconius melpomene*. Genetics 117, 417–426 17603110 10.1534/genetics.107.073122 PMC2013725

[42] Baxter S. W. Papa R. Chamberlin N. Humprey S. J. Joron M. Morrison C. ffrench-Constant R. H. McMillan W. O. Jiggins C. D. 2008 Convergent evolution in the genetic basis of Müllerian mimicry in *Heliconius* butterflies. Genetics 180, 1567–1577 10.1534/genetics.107.082982 (doi:10.1534/genetics.107.082982) 18791259 PMC2581958

[43] Edwards A. W. F. 1972 Likelihood. Cambridge, UK: Cambridge University Press

[44] Chamberlain N. Hill R. I. Kapan D. D. Gilbert L. E. Kronforst M. R. 2009 Polymorphic butterfly reveals the missing link in ecological speciation. Science 326, 847–850 10.1126/science.1179141 (doi:10.1126/science.1179141) 19892982 PMC2875868

[45] Salazar C. A. Jiggins C. D. Arias C. Tobler A. Bermingham E. Linares M. 2005 Hybrid incompatibility is consistent with a hybrid origin of *Heliconius heurippa* Hewitson from its close relatives, *Heliconius cydno* Doubleday and *Heliconius melpomene* Linnaeus. J. Evol. Biol. 18, 247–256 10.1111/j.1420-9101.2004.00839.x (doi:10.1111/j.1420-9101.2004.00839.x) 15715831

[46] Brown K. Emmel T. Eliazar P. Suomalainen E. 1992 Evolutionary patterns in chromosome numbers in neotropical Lepidoptera. I. Chromosomes of the Heliconiini (Family Nyphalidae: subfamily Nymphalinae). Hereditas 117, 109–125 10.1111/j.1601-5223.1992.tb00165.x (doi:10.1111/j.1601-5223.1992.tb00165.x) 1459855

[47] Qvarnströn A. Bailey R. I. 2009 Speciation through evolution of sex-linked genes. Heredity 104, 4–15 10.1038/hdy.2008.93 18781167

[48] Noor M. A. F. Coyne N. 1996 Genetics of a difference in cuticular hydrocarbons between *Drosophila pseudoobscura* and *D. persimilis*. Genet. Res. 68, 117–123 10.1017/S0016672300034005 (doi:10.1017/S0016672300034005) 8940900

[49] Williams M. A. Blouin A. G. Noor M. A. F. 2001 Courtship songs of *Drosophila pseudoobscura* and *D. persimilis*. II. Genetics of species differences. Heredity 86, 68–77 10.1046/j.1365-2540.2001.00811.x (doi:10.1046/j.1365-2540.2001.00811.x) 11298817

[50] Jiggins C. D. Salazar C. A. Linares M. Maváraz J. 2008 Hybrid speciation in *Heliconius* butterflies. Phil. Trans. R. Soc. B 363, 3047–3054 10.1098/rstb.2008.0065 (doi:10.1098/rstb.2008.0065) 18579480 PMC2607310

[51] Melo M. C. Salazar C. A. Jiggins C. D. Linares M. 2008 Assortative mating preferences among hybrids offers a route to hybrid speciation. Evolution 63, 1660–1665 10.1111/j.1558-5646.2009.00633.x (doi:10.1111/j.1558-5646.2009.00633.x) 19492995

